# Tinnitus-related distress after multimodal treatment can be characterized using a key subset of baseline variables

**DOI:** 10.1371/journal.pone.0228037

**Published:** 2020-01-30

**Authors:** Uli Niemann, Benjamin Boecking, Petra Brueggemann, Wilhelm Mebus, Birgit Mazurek, Myra Spiliopoulou

**Affiliations:** 1 Faculty of Computer Science, Otto von Guericke University Magdeburg, Universitaetsplatz 2, Magdeburg, Germany; 2 Tinnitus Center, Charité Universitaetsmedizin Berlin, Charitéplatz 1, Berlin, Germany; Universiteit Antwerpen, BELGIUM

## Abstract

**Background:**

Chronic tinnitus is a complex condition that can be associated with considerable distress. Whilst cognitive-behavioral treatment (CBT) approaches have been shown to be effective, not all patients benefit from psychological or psychologically anchored multimodal therapies. Determinants of tinnitus-related distress thus provide valuable information about tinnitus characterization and therapy planning.

**Objective:**

The study aimed to develop machine learning models that use variables (or “features”) obtained before treatment to characterize patients’ tinnitus-related distress status after treatment. Whilst initially all available variables were considered for model training, the final model was required to achieve highest predictive performance using only a small number of features.

**Methods:**

1,416 tinnitus patients (decompensated tinnitus: 32%) who completed a 7-day multimodal treatment encompassing tinnitus-specific components, CBT, physiotherapy and informational counseling were included in the analysis. At baseline, patients were assessed using 205 features from 10 questionnaires comprising sociodemographic and clinical information. A data-driven workflow was developed consisting of (a) an initial exploratory correlation analysis, (b) supervised machine learning to predict tinnitus-related distress after treatment (T1) using baseline data only (T0), and (c) post-hoc analysis of the best model to facilitate model inspection and understanding. Classification methods were embedded in a feature elimination wrapper that iteratively learned on features found to be important for the model in the preceding iteration, in order to keep the performance stable while successively reducing the model complexity. 10-fold cross-validation with area under the curve (AUC) as performance measure was implemented for model generalization error estimation.

**Results:**

The best machine learning classifier (gradient boosted trees) can predict tinnitus-related distress in T1 with AUC = 0.890 using 26 features. Subjectively perceived tinnitus-related impairment, depressivity, sleep problems, physical health-related impairments in quality of life, time spent to complete questionnaires and educational level exhibited a high attribution towards model prediction.

**Conclusions:**

Machine learning can reliably identify baseline features recorded prior to treatment commencement that characterize tinnitus-related distress after treatment. The identification of key features can contribute to an improved understanding of multifactorial contributors to tinnitus-related distress and thereon based multimodal treatment strategies.

## Introduction

Tinnitus refers to an audiological phenomenon in which a patient perceives a phantom sound (such as ringing, whistling, hissing or rustling) in absence of an external sound source [1]. Tinnitus is a worldwide health problem, with prevalence rates ranging between 12% and 30% [2]. Besides potential hearing loss [3], chronic tinnitus is frequently associated with concomitant psychological difficulties, including depression [4–6], anxiety [5, 7], other somatoform symptoms [8, 9] and insomnia [10]. Cognitive-behavioral approaches have been shown to be effective in the treatment of chronic tinnitus [8, 11–13]. Amongst these, a multimodal tinnitus-specific therapy program has been shown to be effective at 3 and 5-year follow up [14].

Upon presenting at an outpatient clinic, tinnitus patients, including patients suffering from chronic tinnitus who provided the data used in the study, undergo comprehensive medical and psychological assessments that inform individual case conceptualizations and treatment planning. Whilst complex psychobiological interactions are known to contribute to tinnitus-related distress in chronic presentations, reliable and valid assessment procedures can be time-consuming and cumbersome. To reduce patient burden without compromising on assessment validity, it is thus desirable to identify and measure selected key features that are predictive of tinnitus-related distress.

The present paper addresses the following research questions:

Q1 To what extent do baseline features allow for a prediction of tinnitus-related distress *after* multimodal treatment?
Q2 Which features are predictive of tinnitus-related distress before and after treatment completion?
Q3 How many baseline features are necessary for a good prediction of tinnitus-related distress after treatment?

Here, we present a data-driven workflow, which encompasses data preparation steps, machine learning algorithms for the separation of *compensated* and *decompensated* in tinnitus patients, an iteratively invoked module that reduces the feature space while sustaining separation quality, as well as post-hoc interpretation techniques to identify the most important predictors from the trained models.

## Materials and methods

**Fig 1:**
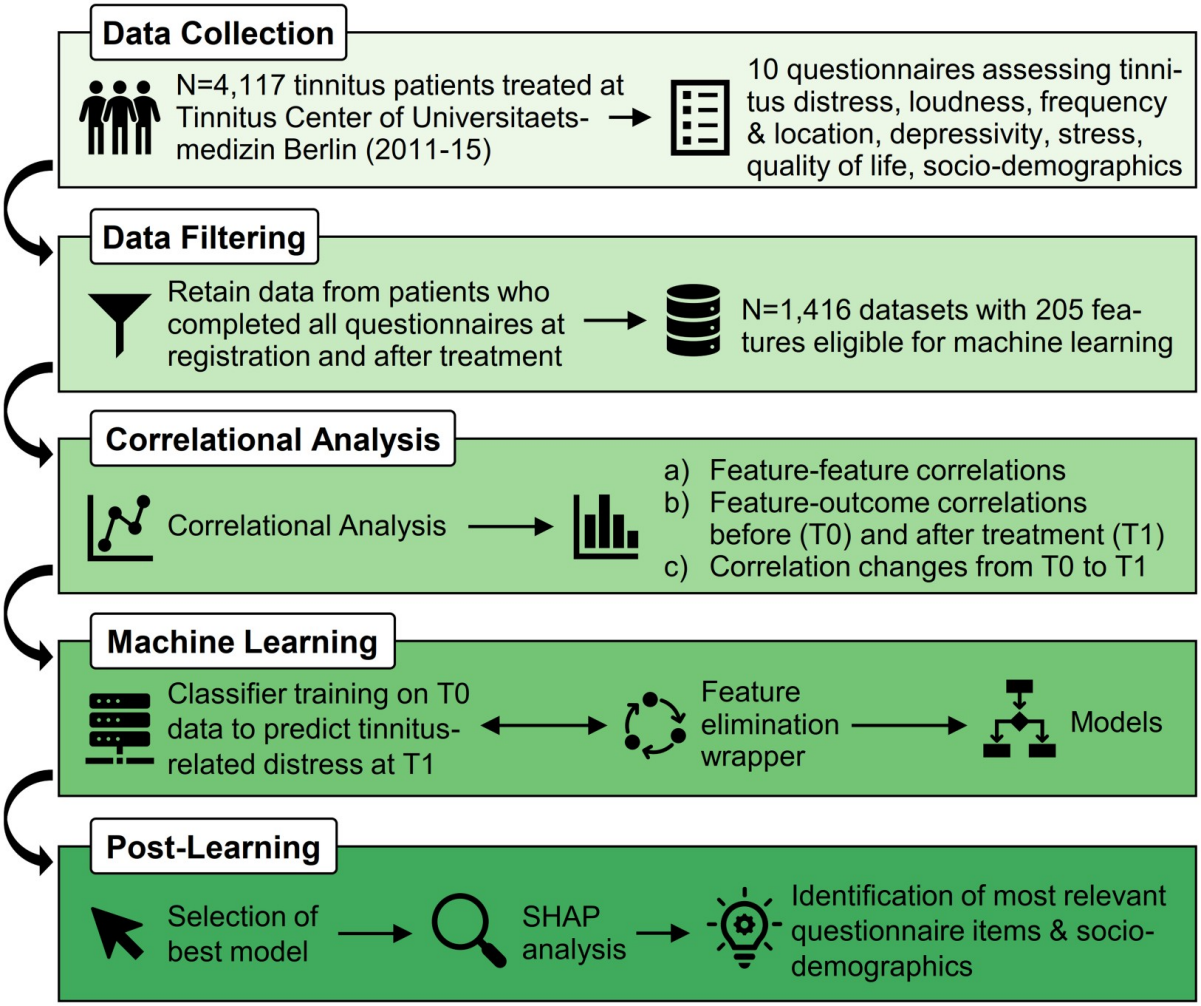
The study workflow. We extracted a total of 205 features from 1,416 patients’ answers to 10 questionnaires. An initial correlational analysis was conducted. Multiple classification models were trained to predict tinnitus-related distress after treatment (T1) using data collected upon baseline (T0). Model training was embedded in an incremental feature elimination wrapper which retained only features which were identified to be important for the model learned at each iteration. Finally, the best overall model (AUC) was selected and the most relevant features were studied further.

Our study workflow is schematically presented in Fig 1, depicting the five main phases (i) data collection, (ii) data filtering, (iii) exploratory correlation analysis, (iv) machine learning including classifier training embedded in an incremental feature elimination wrapper, and (v) post-learning involving model selection and identification of most important features.

### Data collection and filtering

We used data from a cohort of 4,117 tinnitus patients who had been treated at the Tinnitus Center of Charité Universitaetsmedizin Berlin between January 2011 and October 2015. All included patients had been suffering from tinnitus for 3 months or longer, were 18 years of age or older and had sufficient knowledge of the German language. Treatment comprised an intensive, multimodal 7-day program that included informational counseling, detailed ear-nose-throat (ENT) as well as psychological diagnostics, cognitive-behavior therapy interventions, relaxation exercises and physiotherapy. Ethical approval was granted by Charité Universitaetsmedizin Berlin ethics committee (reference number EA1/115/15) and informed written consent was received from all patients. All relevant guidelines and regulations were followed. Prior to the analyses, all data had been anonymized.

A total of 205 features were extracted at baseline (T0) and after treatment (T1), comprising answers to single questionnaire items, subscale scores and total scores from the following 10 questionnaires: (a) General Depression Scale—long form (Allgemeine Depressionsskala; ADSL) [15, 16], (b) Berlin Complaint Inventory (Berliner Beschwerdeinventar; BI) [17], (c) Berlin Mood Questionnaire (Berliner Stimmungsfragebogen; BSF) [18], (d) Perceived Stress Questionnaire (PSQ) [19], (e) Short Form-8 Health Survey (SF8) [20], (f) a sociodemographics questionnaire (SOZK) [21], (g) Self-Efficacy- Optimism-Pessimism Scale (Selbstwirksamkeits-Optimismus-Pessimismus Skala; SWOP) [22], (h) visual analogue scales measuring tinnitus loudness, frequency and distress (TINSKAL) and the (i) Tinnitus Localization and Quality questionnaire (TLQ) [23]. Tinnitus-related distress was assessed using the German version of the (j) Tinnitus Questionnaire (TQ) [24]. Furthermore, for each questionnaire, the average time needed to fill-in an item was recorded. Most questionnaire items comprised multiple-choice questions with answers on a Likert scale. The corresponding ordinal features were handled as continuous features in the analysis. Categorical features, e.g. sex, marital status and education level were encoded as dichotomous features. A brief overview of all features is provided in S1 Table. 1,416 patients (34.4%) who completed all 10 questionnaires before and after treatment were included in the analyses. Table 1 depicts baseline characteristics of all included patients. The TQ tinnitus-related distress score [24] was discretized using the cutoff value of 46 [24] distinguishing between “compensated” (0-46) and “decompensated” tinnitus (47-84). The associated feature TQ_distress measured after treatment (T1) was used as target feature variable for all analyses.

**Table 1:** Baseline characteristics of patients (data at T0).

		Tinnitus-related distress			
		Compensated	Decompensated		
	Total	TQ_distress ≤ 46	TQ_distress > 46	p-value	
Number of subjects, n (%)	1416 (100)	962 (68)	454 (32)		
Age in years	49.8 ± 12.2	49.3 ± 12.4	50.8 ± 11.6	0.025^*TT*^	
Male sex, n (%)	695 (49)	486 (51)	209 (46)	$0.129{}^{\chi^{2}}$	
Tinnitus duration in years, modus (%)	5 (33)	5 (33)	5 (35)	0.012^*MW*^	
ADSL depression score	18.0 ± 11.6	13.7 ± 9.3	27.1 ± 10.8	<0.001^*MW*^	*
BI complaint score	24.5 ± 15.0	19.7 ± 12.5	34.5 ± 15.0	<0.001^*MW*^	*
BSF anger score	0.8 ± 0.7	0.6 ± 0.6	1.2 ± 0.8	<0.001^*MW*^	*
PSQ stress score	0.5 ± 0.2	0.4 ± 0.2	0.6 ± 0.2	<0.001^*TT*^	*
SF8 general health score	41.7 ± 7.1	43.6 ± 6.5	37.7 ± 6.6	<0.001^*MW*^	*
SF8 mental health score	42.0 ± 11.0	45.5 ± 9.8	34.7 ± 9.8	<0.001^*MW*^	*
TQ tinnitus-related distress score	38.3 ± 17.1	28.9 ± 10.9	58.3 ± 8.2	<0.001^*TT*^	*

Baseline characteristics of patients with compensated or decompensated tinnitus respectively. Continuous features are expressed as mean ± standard deviation. Categorical features are expressed as absolute frequency (percentage). P-values were calculated using unpaired two-tailed t-test^*TT*^, $\text{Chi}-\text{squared}\;{\text{test}}^{\chi^{2}}$ or two-tailed unpaired Mann-Whitney test^*MW*^. The significance level was set to *α* = 0.05. An asterisk * indicates statistical significance after Bonferroni correction of the critical value, i.e., *p*_*crit*_ = *α*/number of comparisons = 0.05/10 = 0.005. ADSL: general depression scale—long form [15, 16]; BI: Berlin Complaint Inventory [17]; BSF: Berlin Mood Questionnaire [18]; PSQ: Perceived Stress Questionnaire [19]; SF8: Short-form 8 Health Survey [20]; TQ: German version of the tinnitus questionnaire [24].

### Exploratory correlational analysis

Exploratory correlational analysis assessed the strength of bivariate relationships among the recorded features at T0 as well as between each feature and the TQ distress score. More specifically, the Spearman correlation coefficient was calculated (i) to identify groups of features with similar intra-group and inter-group correlations, (ii) to assess questionnaire median correlations with the TQ distress score measured in T0 and T1, (iii) to identify features with the highest correlational magnitude with respect to the TQ distress score in T0 and T1 and (iv) to identify features whose correlational effects with the TQ distress score differed between T1 and T0.

### Classifier training

The potential of machine learning for the prediction of TQ_distress at T1 using questionnaire data from T0 only was investigated with the following 11 algorithms: LASSO [25], RIDGE [26], generalized partial least squares (GPLS) [27], support vector machine (SVM) [28], a feed-forward neural network with one single hidden layer (NNET) [29], weighted k-nearest neighbor classifier (WKNN) [30], Naïve Bayes classifier (NB), CART decision tree [31], C5.0 decision tree [32], random forest (RF) [33] and gradient boosted trees (GBT) [34].

- LASSO and RIDGE are extensions of ordinary least squares linear regression. Their objective function contains an additional penalty term, either to control the inclusion of predictors (LASSO) or to shrink the magnitudes of the regression coefficients (RIDGE). As a result, models tend to have better predictive performance with better interpretability due to their inbuilt feature subset selection in comparison with ordinary linear regression.
- Partial least squares is another extension of linear regression which first performs a dimension reduction by constructing a new set of features that are linear combinations of the original features, and then fit a linear regression to these new features. Often, the number of features of the projection is set to be much lower than the number of features of the original feature space.
- SVMs are capable of modeling non-linear relationships between the predictors and the target feature. They use a non-linear mapping to enlarge the feature space of the original training data into a higher dimension. Within this new dimension, the optimal linear separating *hyperplane* is identified. This hyperplane is the decision boundary separating the observations from different classes.
- NNET is an example of neural networks. Neural networks extract new features by linear combinations of the original features and to use them to model the target feature as non-linear function of these features.
- WKNN is a variant of the KNN classification algorithm which does not build a model at all, but rather identifies for a particular observation with unknown class label the K “closest” observations from the training data and uses their majority class as prediction. Closeness is defined by a distance measure such as the Euclidean distance. WKNN uses distance to weight the influence a training observation has on the prediction, such that training instances with low distance obtain a higher weight.
- NB uses Bayes’ theorem to calculate class membership probabilities. The “naive” property refers to the assumption of class-conditional independence among the features, which is employed to reduce computational complexity.
- CART, C5.0, RF and GBT are tree-based methods. Classifiers of this family partition the feature space into a set of non-overlapping rectangles based on combinations of feature-value conditions, such as “IF age < 52 & ADSL_depression > 19”. To make a prediction for a given observation, they use the majority class of training data assigned to the rectangle it belongs. Random forests and gradient boosted trees are ensembles of different simple decision trees, where each tree casts a vote towards the final prediction. Whereas trees in a random forest are built independently from each other, boosted trees iteratively add a tree to the composite model, aiming to reduce the classification error of the previously learned set of trees.

10-fold stratified cross-validation was used for classifier evaluation. In k-fold cross-validation, the data is split into k partitions. Each partition serves once as test set for the model which is trained on the remainder of the partitions. Finally, the k performance results are averaged. A grid search was employed for algorithm hyperparameter selection (cf. tuning grid in S2 Table). The area under the receiver operating characteristic curve (AUC) was used as performance measure. A receiver operating characteristic curve is a plot that juxtaposes sensitivity (true positive rate (TPR)) and false positive rate (FPR) for varying thresholds of a binary classifier. The area under the ROC curve (AUC) takes values from 0 (0% TPR, 100% FPR) to 1 (100% TPR, 0% FPR). The higher the AUC, the better is the classifier at distinguishing between patients with decompensated and compensated tinnitus.

### Feature elimination

Although some of the utilized classification algorithms are insensitive to a high number of features, there are several reasons to remove superfluous predictors. For example, the selection of a feature subset contributes to the prevention of overfitting, the avoidance of multicollinearity and the identification of a model with good trade-off between high predictive performance and low complexity, i.e., a low number of features. Here, a feature elimination wrapper was developed that iteratively discarded a subset of features which were not contributing to predictive performance. This mechanism is an extension of the feature importance score for random forests [33] and its generalization to any model type [35], referred to as “model reliance”. The model reliance estimates the worth of an individual feature *f* by comparing the classification error on the original training set with the classification error on a modified version of the training set where the values of *f* are randomly permuted. A high model reliance score expresses high dependency of the model prediction on *f*, since the random permutation increased the classification error.

The model reliance *MR* of a model *ζ* on a feature *f* ∈ *F* is calculated as follows: First, the classification error on the original training data is calculated as *e*_*orig*_ = *CE*(*y*, *ζ*(**X**_*orig*_)), where *CE* is the classification error function, *y* is the target feature and *ζ*(**X**_*orig*_) is predicted target feature on the original training data. Then, the values of *f* are randomly permuted and the classification error on the slightly modified dataset **X**_*perm*_ is calculated as *e*_*perm*_ = *CE*(*y*, *ζ*(**X**_*perm*_)). Finally, the model reliance *MR*(*f*) is the ratio of the two terms, i.e., $MR\left(f,\zeta \right)=\frac{e_{perm}}{e_{orig}}$. Since feature perturbation introduces a degree of randomness, *MR* was calculated as average over 10 runs as a more stable estimate. A *MR* value greater below 1 suggests that *f* is adversarial to model performance. Thus, our feature elimination wrapper successively removes these features to then train a new classifier on the subset of predictors with *MR* > 1. In the first iteration *i* = 1, an initial model *ζ*_1_ is calculated on the full feature set *F*_1_ = *F*. For each feature, the model reliance *MR*(*f*, *ζ*_*i*_) is calculated. Features with *MR*(*f*, *ζ*_*i*_) > 1 are retained for iteration *i* + 1 while the remaining features are dropped. This procedure continues until either none of the *MR* values exceed 1, i.e., ∀*f* ∈ *F*_*i*_: *MR*(*f*, *ζ*_*i*_) ≤ 1, or the feature set in iteration *i* is identical to the feature set in iteration *i* − 1, i.e., *F*_*i*_ = *F*_*i*−1_.

### Feature importance

Understanding the prediction of a classification model is a major challenge in order to obtain actionable insights that can ultimately contribute to improve prevention, diagnosis and treatment. Many state-of-the-art algorithms such as gradient boosted trees produce models with high accuracy. However, these so called black-box models are complex and not intrinsically understandable as they usually incorporate many multi-variate, non-linear relationships among groups of features, which are hard to present to the domain expert intuitively. A trade-off between predictive quality and understandability often means using less complex methods such as linear models and decision trees. Instead, in this study, both complex and understandable classifiers were investigated. To facilitate model interpretation, the model-agnostic post-hoc framework SHAP [36, 37] was used to assess feature importance. Briefly, the SHAP value *ϕ*_*f*_(*ζ*, *x*) expresses the estimated importance of a feature *f* to the prediction of model *ζ* for an instance *x* as change in the expected value of the prediction if for *f* the feature vector of *x* is observed instead of being random. The SHAP framework composes the model prediction as sum of SHAP values of each feature, i.e., $\zeta \left(x\right)=\phi_{0}\left(\zeta ,x\right)+\sum_{i=1}^{M}\phi_{i}\left(\zeta ,x\right)$, where *ϕ*_0_(*ζ*, *x*) is the expected value of the model (bias) and *M* is the number of features.

SHAP values were calculated for the best model *ζ*_*opt*_ according to AUC. A ranking of T0 feature attribution towards *ζ*_*opt*_ was determined by calculating the average SHAP value magnitude over all instances, i.e., $A\left(j\right)=\sum_{i=1}^{N}\left|\phi_{j}\left(\zeta_{opt},x\right)\right|$, where *A*(*j*) is the attribution of the *j*-th feature. The *N* × *M* SHAP matrix was clustered with agglomerative hierarchical clustering to identify subgroups of patients with similar SHAP values.

## Results

### Distribution of tinnitus-related distress at T0 and T1

**Fig 2:**
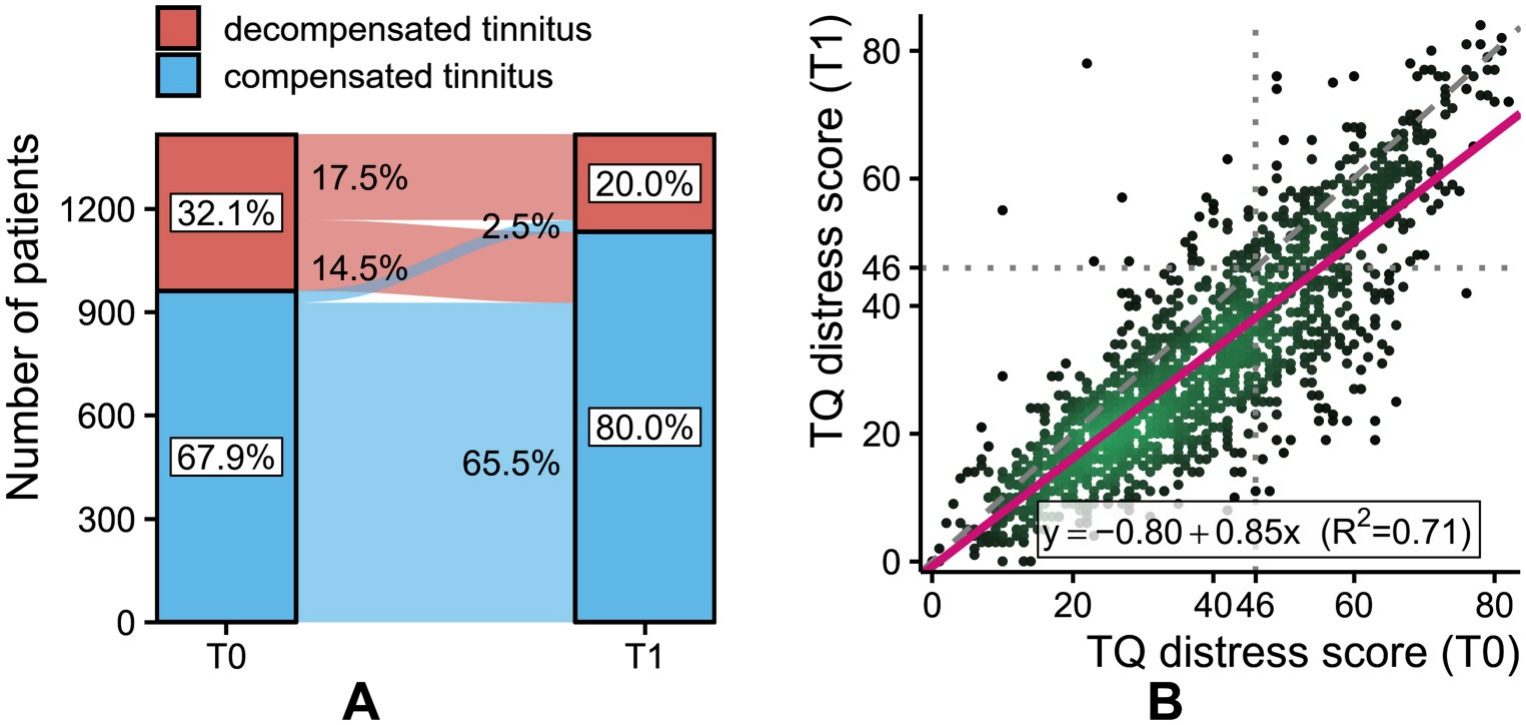
Tinnitus-related distress (TQ_distress) before and after treatment. (A) 83% of patients did not move between categories of overall tinnitus-related distress status (i.e. compensated or decompensated) with treatment. However, significantly more patients transitioned from “decompensated” to “compensated” tinnitus (14.5%) than vice versa (2.5%). (B) The slope of the regression line indicates a general decrease in tinnitus-related distress as a result of treatment.

Approximately a third (32.1%) of the 1,416 subjects reported decompensated tinnitus at T0 (Fig 2A). Almost half of these patients (14.5%) transitioned to compensated tinnitus (CT) with treatment. Overall, 283 out of 1,416 patients (20.0%) showed decompensated tinnitus at T1. A general positive effect of treatment is indicated by the slope of the linear regression line below 1.0 in Fig 2B with TQ_distress in T1 as dependent variable and TQ_distress in T0 as independent variable.

### Correlation analysis

**Fig 3:**
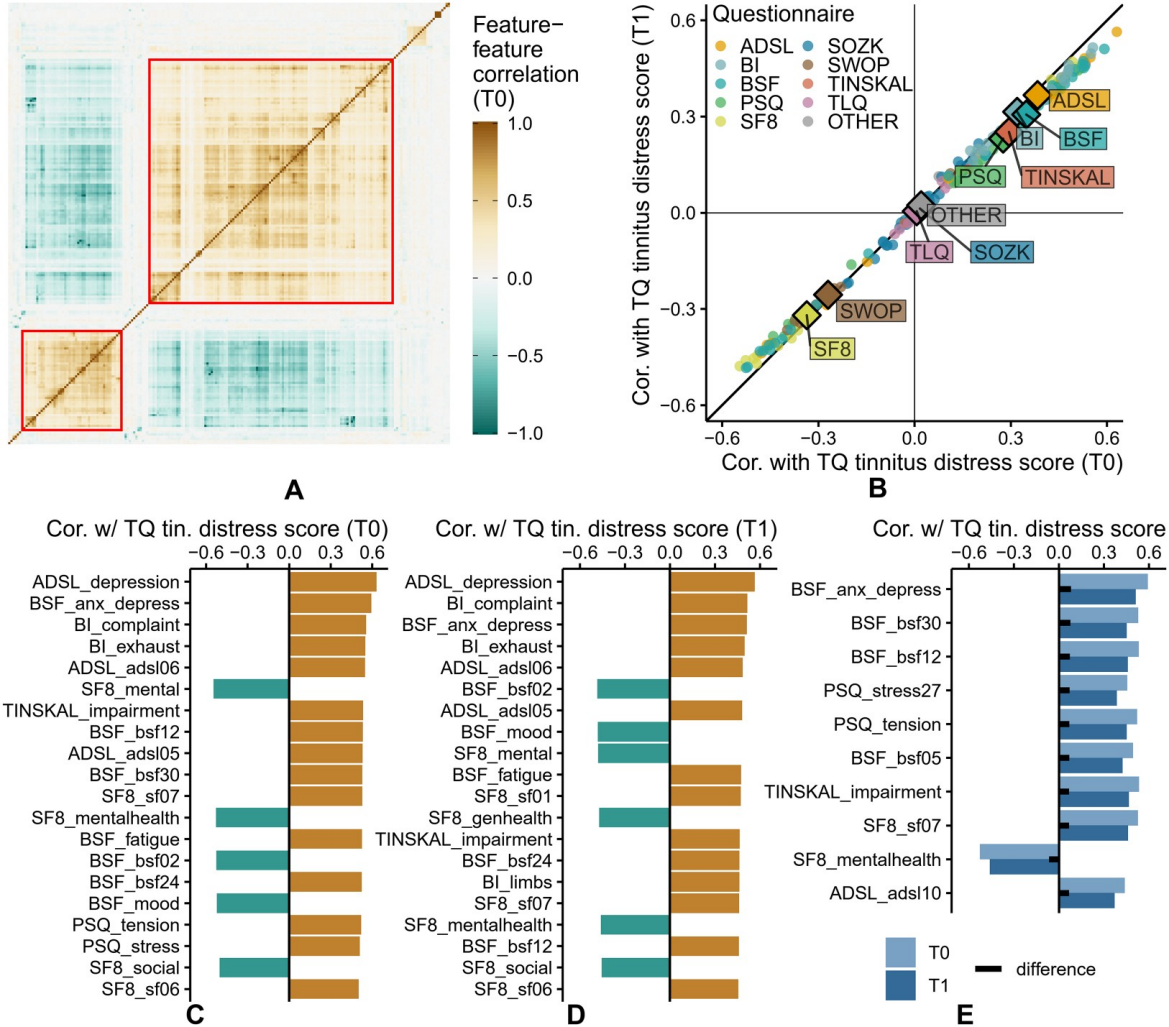
Feature-feature correlation & feature correlation with respect to TQ tinnitus-related distress score in T0 and T1. (A) Correlation heatmap for all pairs of features (T0). Features are ordered by agglomerative hierarchical clustering with complete linkage. (B) Correlation of each feature with TQ tinnitus-related distress score, in T0 (x-axis) and in T1 (y-axis). The diamond symbol represents a questionnaire’s median. (C) Top-20 features with highest correlation to TQ tinnitus-related distress score (T0). (D) Top-20 features with highest correlation to TQ tinnitus-related distress score (T1). (E) Top-10 features whose correlational effects with TQ tinnitus-related distress score differ in T0 vs. T1. Correlation values before and after treatment are shown as light blue and dark blue bars, respectively. Differences in correlation are represented as black bars centered in between.

The heatmap in Fig 3A shows all pairwise feature-feature correlations in T0. Two major feature groups were identified, indicated by red squares, with moderate to high intra-group and negative inter-group correlations. The upper group (cf. Fig 3A) comprised 114/205 features (55.6%) representing negatively polarized questionnaire items and scales, e.g. the ADSL depressive disorder score (ADSL_depression) and the BI complaint strain score (BI_complaint). Conversely, the lower group included 47/205 positively polarized features (22.9%), e.g. the SF8 mental health score (SF8_mental) and the BSF elevated mood score (BSF_mood). Fig 3B juxtaposes pairwise correlations of each feature and TQ tinnitus-related distress score *before* (x-axis) and *after* treatment (y-axis). No strong bivariate correlation was observed, as all values lay within the interval -0.6 and 0.6. The average change in absolute correlations between the beginning and end of treatment was 0.031. The change in absolute correlation was less than 0.067 for 95% of the features (cf. closeness of the points to the diagonal line in Fig 3B). For 137 out of 205 features (66.8%), the absolute value of correlation decreased from T0 to T1. Median correlations of the questionnaires ADSL, BSF and BI (SF8) were above (below) 0.3 (-0.3) at both moments, respectively, and thus higher than for the remaining questionnaires. Fig 3C and 3D reveal that features from 6 questionnaires were among the top-20 features ranked by absolute correlation with TQ_distress in T0 and T1. The general depression score ADSL_depression showed strongest correlation before (*ρ* = 0.630) and after treatment (*ρ* = 0.564). Fig 3E depicts the 10 features with the largest differences in correlation magnitudes after vs. before treatment. For each of these features, the correlation before treatment is higher.

### Predictive performance of classification models

The classification models predicted tinnitus-related distress compensation after treatment based on questionnaire answers and socio-demographic data acquired at baseline with high AUC. Table 2 depicts the performance of all 11 classification methods across 10 feature elimination rounds. The gradient boosted trees model (GBT) achieved highest AUC (iteration i = 7, AUC = 0.890±0.04 [0.887,0.893]; mean±SD [95% CI]), using only 26 features. The RIDGE classifier yielded second-best performance (i = 2, AUC: 0.876±0.05 [0.873,0.879]), relying on 127 features, followed by the random forest model (i = 3, AUC: 0.872±0.05 [0.869,0.875]) using 77 features. Classification using the best model (GBT, i = 7) based on a probability threshold of 0.5 resulted in an accuracy of 0.86, a true positive rate (sensitivity) of 0.72, a true negative rate (specificity) of 0.88, a precision of 0.48 and a negative predictive value of 0.95.

**Table 2:** Average cross-validation AUC and number of features (p) for each classifier with optimal hyperparameter configuration and for each feature selection iteration (i). For each classifier, the best AUC is highlighted in boldface. Classifiers are ordered by their maximum AUC. All methods induced at least one model with AUC of 0.790 or higher. Cells with a “/” indicate that the feature elimination wrapper had already been terminated after a previous iteration.

	*i* = 1		*i* = 2		*i* = 3		*i* = 4		*i* = 5		*i* = 6		*i* = 7		*i* = 8		*i* = 9		*i* = 10	
Classifier	AUC	p	AUC	p	AUC	p	AUC	p	AUC	p	AUC	p	AUC	p	AUC	p	AUC	p	AUC	p
GBT	.880	205	.885	134	.885	90	.883	59	.888	43	.890	31	**.890**	26	.889	24	.887	21	/	/
RIDGE	.873	205	**.876**	127	.870	85	.858	32	.859	19	.856	11	.858	10	.831	6	.782	2	/	/
RF	.866	205	.870	109	**.872**	77	.872	54	.871	48	.870	44	.871	22	/	/	/	/	/	/
LASSO	.869	205	**.872**	103	.871	52	.870	25	.856	14	.857	8	/	/	/	/	/	/	/	/
SVM	.864	205	**.871**	84	.863	38	.868	28	.864	21	.865	15	.865	13	.862	9	/	/	/	/
WKNN	**.848**	205	.834	67	.817	31	.825	15	.823	11	.811	7	/	/	/	/	/	/	/	/
GPLS	.830	205	**.842**	98	.841	57	.838	21	.835	5	/	/	/	/	/	/	/	/	/	/
NNET	.780	205	.823	108	.812	67	.811	46	.798	32	.811	30	.822	28	**.827**	21	.827	18	.811	17
NB	.822	205	**.826**	99	.795	35	.800	18	.786	7	.781	6	.791	5	/	/	/	/	/	/
CART	.778	205	.789	93	.785	50	.796	35	.794	25	.798	22	.797	21	.797	20	**.800**	19	/	/
C5.0	.764	205	.755	106	.754	68	.753	46	.760	30	.768	25	**.790**	16	.784	14	/	/	/	/

### Classifier performance on smaller feature spaces

When trained using a smaller feature space, each classifier produced at least one model with nearly equal or even improved performance compared to the respective model learned on the whole feature space. In fact, except for WKNN, all classification methods benefited from feature elimination as they produced the best model on a reduced feature space (cf. Table 2). For GBT, the increase in AUC from 185 features to 26 features (i = 11) was 0.01. This model achieves both maximum AUC and a well-balanced trade-off between high predictive performance and low model complexity, and we thus decided to further investigate this model.

### Feature importance

**Fig 4:**
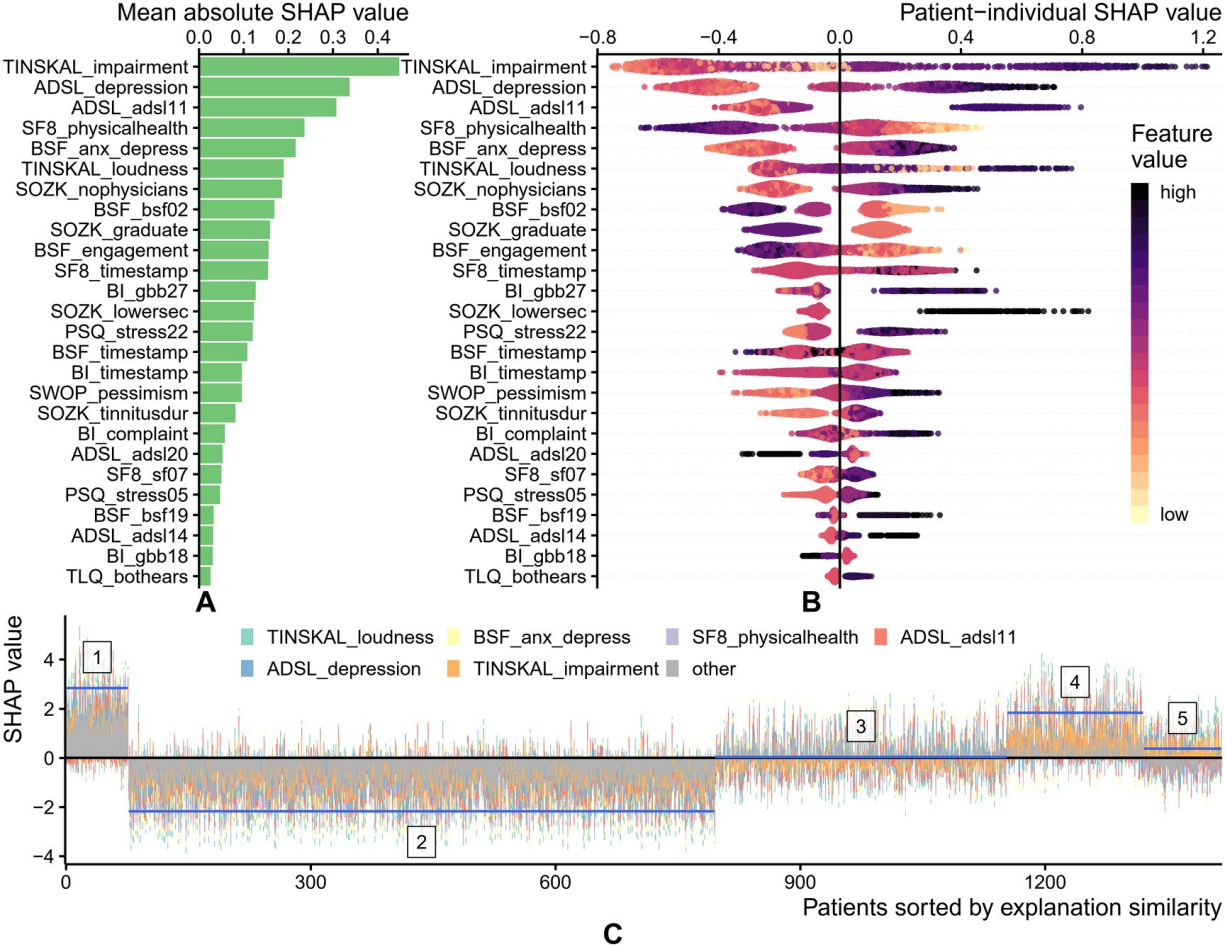
SHAP analysis results for the best model (GBT, i = 7). (A) Global feature importance based on the mean absolute magnitude of the SHAP values over all training instances. Values represent absolute change in log odds where higher values indicate higher feature importance. (B) Instance-individual SHAP values. A point represents the SHAP value for the feature depicted on the y-axis with respect to a single patient. The further afar a point from the vertical line at 0.0, the larger the attribution of the corresponding feature value to the model prediction. Vertically offset points depict regions of high density. Points are colored according to the actual feature value of the respective patient. (C) Combined SHAP feature attribution for all patients. Patients are ordered according to hierarchical clustering with complete linkage and *k* = 5. Blue horizontal lines depict the average sum of SHAP values of the cluster members.

**Fig 5:**
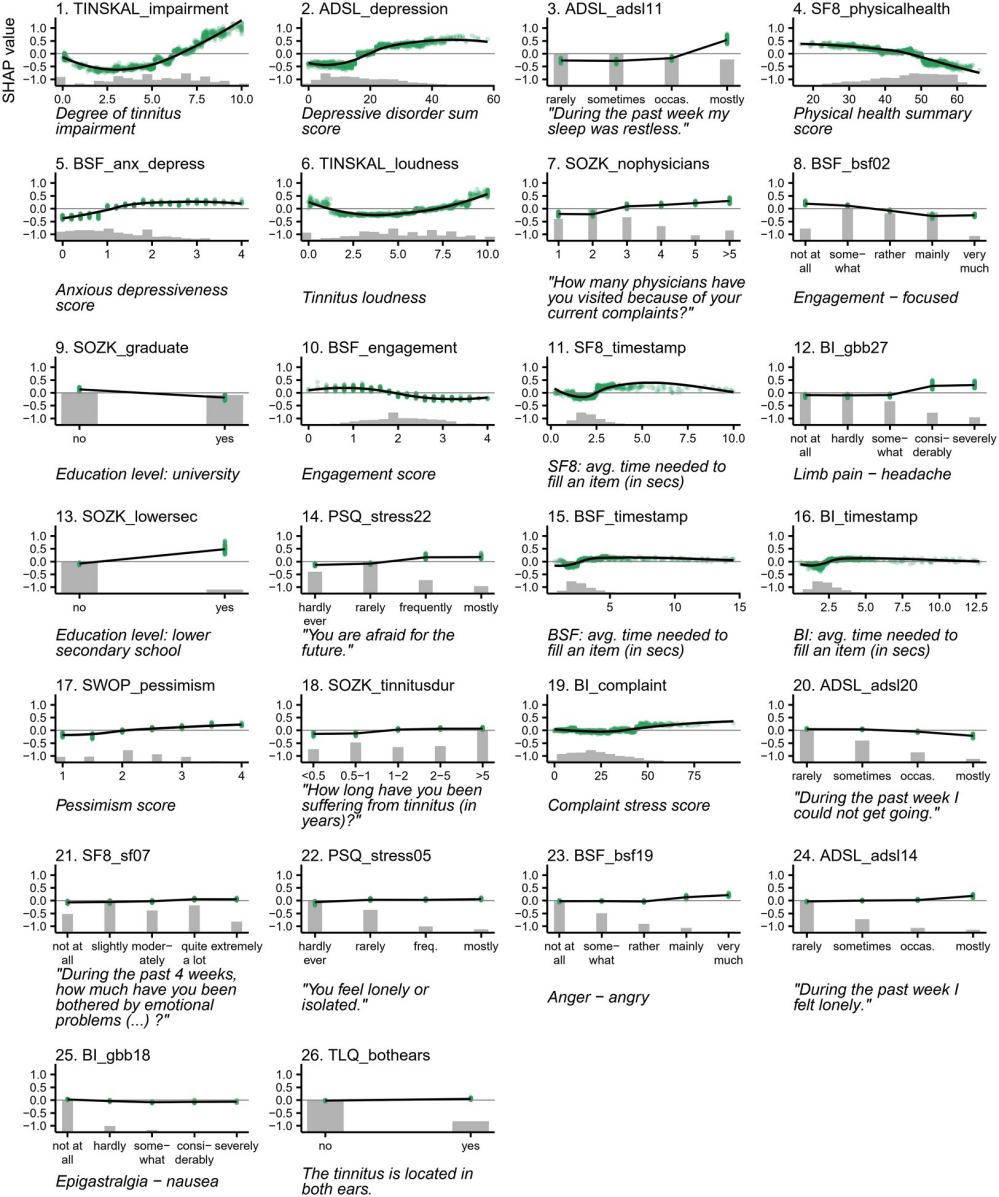
SHAP feature dependence plots. The relationship between actual feature values (x-axis) and corresponding SHAP values (y-axis) is shown as green points. Positive SHAP values indicate an increased risk of decompensated tinnitus relative to the training set average, and vice versa. A locally weighted scatterplot smoothing (LOWESS) is placed as black curve on top to indicate the global trend. Gray bottom histograms represent the distribution of the actual feature values.

For the best model, the attributions of the 26 selected features are shown in Fig 4A. The TINSKAL impairment score (TINSKAL_impairment) was identified as most important, with an average absolute SHAP value magnitude (change in log odds) of 0.448. The ADSL depression score (ADSL_depression) and a single question from the general depression score questionnaire (ADSL_adsl11: “During the past week my sleep was restless.”) emerged as second and third most relevant. Besides 6 aggregated (sub-)scores, 12 single questionnaire items, 4 socio-demographic features (number of visited doctors, university-level education, lower secondary education, duration of tinnitus) and 4 features indicating time spent filling the questionnaires were selected. Notably, at least 1 feature from each of the 9 questionnaires was chosen. Fig 4B depicts the patient-individual SHAP values for each feature as points where color represents the actual feature value. The high attribution of TINSKAL_impairment is emphasized by the wide spread in the value distribution. For this feature, high feature values correspond to an increased probability of tinnitus decompensation. However, this trend is not monotone, since small values (light orange) are associated with a SHAP value just slightly below 0. This is emphasized in the feature dependence plots in Fig 5 which juxtapose feature values and corresponding SHAP values for all patients and for each of the 26 selected features. In fact, the top-left feature dependence plot in Fig 5 reveals a curvilinear, J-shaped relationship between TINSKAL_impairment and SHAP values. More specifically, the predicted tinnitus-related distress decreases within the range [0,3] with increasing values, whereas it decreases within (3,10]. Similar patterns were observed for ADSL_depression, TINSKAL_loudness, BI_complaint, BSF_timestamp and SWOP_pessimism. Although some of the features are not highly ranked globally, their impact on specific subgroups is high. For example, the feature SOZK_lowersec (lower secondary education) considerably increases the risk of tinnitus-related distress for patients with lower secondary education, but only marginally reduces the risk for patients of other education levels. For the majority of features, the relationship with predicted tinnitus-related distress is monotone. For instance, the SF8 physical score (SF8_physical) shows decreasing predicted tinnitus-related distress with increasing physical health. Fig 4C shows stacked patient-individual SHAP values for the six features with highest average SHAP value magnitude and the combined rest. Five subgroups of patients with similar explanation similarity were identified. Cluster 1 comprises a large fraction of patients with secondary school education (43 out of 76) which is considerably higher than the overall average of 11%. This subgroup has the highest risk of being classified with decompensated tinnitus (blue line depicts cluster average). Cluster 2 is the largest subgroup containing 50.7% of all patients. This subgroup can be characterized with depression severity and an overall low risk of high tinnitus severity. Cluster 4 is described by a high tinnitus impairment (TINSKAL_impairment) whereas clusters 3 and 5 are more heterogeneous with their average prediction close to the prior, respectively. For each of the 26 features with the highest average SHAP value magnitude, Fig 5 shows patient-individual feature values and corresponding attribution towards the best model. TINSKAL_impairment (tinnitus impairment), ADSL_depression (depression severity), TINSKAL_loudness, BSF_engagement and the timestamp features appear to exhibit non-monotonic relationships.

## Discussion

The present study used data from multiple self-report questionnaires acquired at baseline in order to build a classification model for the prediction of tinnitus-related distress in patients with chronic tinnitus following multimodal treatment. The best classifier (gradient boosted trees model) that separated between patients with “compensated” and “decompensated” tinnitus after treatment (T1) with high AUC utilized 26 from a total of 205 features acquired at baseline (T0). While a considerable reduction in dimensionality was achieved by removing approx. 87% of the original features, none of the 9 questionnaires appeared to be negligible for the prediction of tinnitus-related distress, as each questionnaire contributed at least one feature to the optimal feature space.

The best model utilizes features that describe a variety of psychological and psychosomatic patient characteristics as well as socio-demographics thereby confirming the multi-factorial nature of tinnitus-related distress; these characteristics can be used for phenotyping and then for a followup investigation of how such characteristics influence treatment success. As expected, the model attributions of features that are directly linked to tinnitus quality, such as the degree of perceived tinnitus impairment and loudness, appeared to be high. At the same time, depression, attitudinal factors (self-efficacy, pessimism, complain propensity), sleep problems, educational level, tinnitus location and duration emerged as highly important for the model prediction as well.

Qualitative predictors, such as tinnitus impairment (TINSKAL_impairment) and loudness (TINSKAL_loudness), exhibited non-monotonic relationships with respect to the predicted outcome. Notably, very low self-reported impairment or loudness did not indicate a strong improvement in tinnitus-related distress. In future, these findings could be investigated further, e.g., whether there is a relationship towards a subgroup of patients that were more fatigued and thus not less thoroughly filling a large number of questionnaires. Another explanation could be that a simple measurement like TINSKAL_impairment and TINSKAL_loudness is less robust and exhibits higher variability than a compound scale that combines multiple single questionnaire items.

This study once more confirms the intricate interplay between depression and tinnitus-related distress that was emphasized by numerous previous studies [38–42]. For our best model, patients with an ADSL depression score (ADSL_depression) of less than 16 were predicted with a low tinnitus decompensation probability whereas patients exceeding 22 are more likely (cf Fig 5). This cutoff is consistent with the classification of irrelevant and relevant depression from [16].

Some of the features appear to have a higher impact on the model decision for a subset of patients, including the two selected features on educational level (SOZK_graduate and SOZK_lowersec). This finding is consistent with the clustering on patients based on explanation similarity (cf. Fig 4C) which revealed two subgroups (clusters 1 and 4) that are characterized by a lower school degree and high degree of reported tinnitus impairment in comparison with the other clusters, including one large subgroup (50%) that exhibits low depression scores.

### How many features are really necessary for a good tinnitus-related distress prediction?

**Fig 6:**
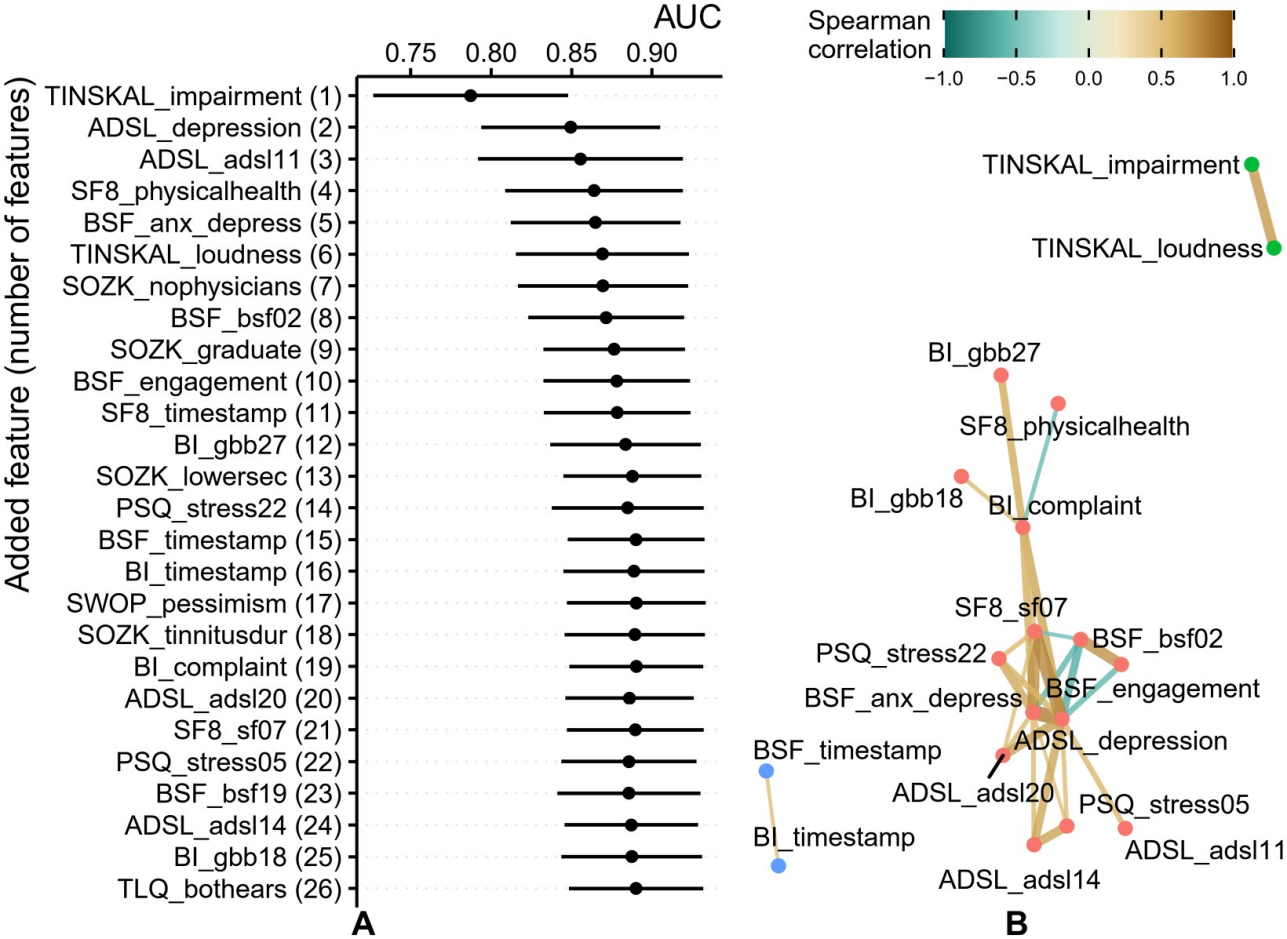
Marginal feature importance. (A) Average cross-validation AUC (± SD) of a row refers to the performance of a GBT model trained on the feature subset that consists of the feature depicted on the y-axis label and all features of the above rows. The ordering of features is according to mean absolute SHAP value magnitude (cf. Fig 4A). (B) Network visualization illustrating 3 groups among the 26 selected features of the best model with high intra-group correlation. 8 features (predominantly SOZK features) without pairwise correlation of magnitude 0.5 or higher were dropped.

Fig 6A illustrates the increase of predictive performance of GBT models when a further feature is successively added to the feature space. A model that uses only the feature TINSKAL_impairment yields an AUC of 0.79±0.06. While adding ADSL_depression leads to an increase in AUC of 0.06, none of the remaining 24 features from the best model makes for a total improvement of more than 0.01, respectively. Only 3 features are necessary to achieve a mean AUC of 0.85, 8 features for a mean AUC of 0.87 and 15 features for a mean AUC of 0.89 (cf. Fig 6A). One potential reason might be a considerable degree of multicollinearity among groups of features. Fig 6B shows a network of 3 feature groups among the 26 features of the best model. For example, the features TINSKAL_impairment and TINSKAL_loudness are moderately correlated (*ρ* = 0.69), which leads to the question whether one of the pair might be omitted without a considerable loss in predictive power. The largest subgroup spanning 14 features involves descriptors of depression, perceived stress and reported physical health. In future work, an investigation of possible interaction effects among these moderately to strongly correlated features could be investigated, to better understand why all of them were selected and to determine whether some of them could be removed to achieve a better trade-off between model accuracy and complexity.

### Strengths and weaknesses of the study

To our knowledge, this is the first study that investigates the potential of machine learning for the identification of most important predictors from a wide range of features acquired before treatment for tinnitus compensation after treatment. The data-driven approach ensures that any potential predictor is included in the analysis. Another strength of the study is the internal validation of the classification models using cross-validation and hyperparameter tuning. Further, due to the selection of a variety of classification algorithm families, both linear as well as non-linear relationships between a feature and the outcome could be identified. A consequent limitation of this hypothesis-free approach is that the learned models could contain features that quantify the same or similar patient characteristics. For example, the best model in this study incorporated the two strongly correlated features ADSL_depression *and* BSF_anx_depression (anxious depressiveness score). Whilst the inclusion of both features was contributing to model performance, from a medical perspective a prediction model with only distinct features might be more beneficial. A pre-selection of features to avoid multicollinearity could be a direction for future work.

Finally, the exclusion of 2,701 out of 4,117 patients (65.6%) who did not complete *all* 10 questionnaires might led to a selection bias. Many patients spent more than one hour filling the questionnaire on a dedicated minicomputer and thus were more likely to abort the completion process, partly due to a gradual loss of motivation to give answers to a large number of questions, technical unfamiliarity with a computer, or interruptions by the hospital staff who demanded to continue with other baseline examinations. Completers were slightly younger than non-completers (mean age 49.8±12.2 vs. 51.7±13.8), were more likely to have the highest German school degree “Abitur” (48.2% vs. 42.0%) and had been suffering from tinnitus longer (> 5 years: 33.3% vs. 25.1%). A detailed comparison between completers and non-completers can be found in S3 Table. To our knowledge, no study has as yet investigated differential treatment responses in completers vs. non-completers; this can be explained by the absence of adequate information on the latter. We intend to investigate to what extent insights from completers can be used on subsamples of non-completers. For this, we can use the DIVA framework of Hielscher et al. [43]. However, psychological treatment approaches are only likely to benefit those reporting psychological distress prior to or associated with the tinnitus percept.

## Conclusion

Our study establishes a first step towards creating a data-driven model for the prediction of tinnitus-related distress based on a small subset of variables extracted from a larger set of baseline questionnaires. From a clinical point of view, the inclusion of features from different questionnaires in the best model indicates the importance of continuing to assess different psychological constructs in order to accurately predict and understand the nature and malleability of tinnitus-related distress. Future work includes the identification of predictive features for treatment response.

## Supporting information

S1 Table Feature overview. A listing of all 205 features that were used for classifier training. These features were extracted at baseline (T0) and after treatment (T1), comprising answers to single questionnaire items, subscale scores and total scores from the following questionnaires: (a) General Depression Scale—long form (Allgemeine Depressionsskala; ADSL) [15, 16], (b) Berlin Complaint Inventory (Berliner Beschwerdeinventar; BI) [17], (c) Berlin Mood Questionnaire (Berliner Stimmungsfragebogen; BSF) [18], (d) Perceived Stress Questionnaire (PSQ) [19], (e) Short Form-8 Health Survey (SF8) [20], (f) a sociodemographics questionnaire (SOZK) [21], (g) Self-Efficacy- Optimism-Pessimism Scale (Selbstwirksamkeits-Optimismus-Pessimismus Skala; SWOP) [22], (h) visual analogue scales measuring tinnitus loudness, frequency and distress (TINSKAL) and the (i) Tinnitus Localization and Quality questionnaire (TLQ) [23].
(PDF)

S2 Table Classifier hyperparameter tuning grid. The potential of machine learning for the prediction of TQ_distress at T1 (after treatment) using questionnaire data from T0 only was investigated with the following 11 algorithms: LASSO [25], RIDGE [26], support vector machine (SVM) [28], a feed-forward neural network with one single hidden layer (NNET) [29], generalized partial least squares (GPLS) [27], weighted k-nearest neighbor classifier (WKNN) [30], Naïve Bayes classifier (NB), CART decision tree [31], C5.0 decision tree [32], random forest (RF) [33] and gradient boosted trees (GBT) [34]. All classifiers were implemented with the statistical programming language R [44] using the package mlr [45], which provides a consistent interface to many machine learning algorithms from other R packages. A grid search was employed for hyperparameter tuning using area under the ROC curve (AUC) as evaluation measure. The table below provides an overview about each classifier, including used R package, tuned hyperparameters and their value ranges. Any other hyperparameters were set to default values.
(PDF)

S3 Table Comparison of completer and non-completer characteristics. Relative frequencies are given in percent.
(PDF)
