# Supplementary material for: Tinnitus-related distress after multimodal treatment can be characterized using a key subset of baseline variables
Source: PLoS One. 2020 Jan 30;15(1):e0228037. doi: 10.1371/journal.pone.0228037 (PMC6991951; doi:10.1371/journal.pone.0228037)
Supplement: S1 Table — A listing of all 205 features that were used for classifier training. These features were extracted at baseline (T0) and after treatment (T1), comprising answers to single questionnaire items, subscale scores and total scores from the following questionnaires: (a) General Depression Scale—long form (Allgemeine Depressionsskala; ADSL) [15, 16], (b) Berlin Complaint Inventory (Berliner Beschwerdeinventar; BI) [17], (c) Berlin Mood Questionnaire (Berliner Stimmungsfragebogen; BSF) [18], (d) Perceived Stress Questionnaire (PSQ) [19], (e) Short Form-8 Health Survey (SF8) [20], (f) a sociodemographics questionnaire (SOZK) [21], (g) Self-Efficacy- Optimism-Pessimism Scale (Selbstwirksamkeits-Optimismus-Pessimismus Skala; SWOP) [22], (h) visual analogue scales measuring tinnitus loudness, frequency and distress (TINSKAL) and the (i) Tinnitus Localization and Quality questionnaire (TLQ) [23]. (PDF) [file pone.0228037.s001.pdf]

S1 Table. Feature overview. A listing of all 205 features that were used for classifier training. These features were extracted at baseline (T0) and after treatment (T1), comprising answers to single questionnaire items, subscale scores and total scores from the following questionnaires: (a) General Depression Scale - long form (Allgemeine Depressionsskala; ADSL) [1, 2], (b) Berlin Complaint Inventory (Berliner Beschwerdeinventar; BI) [3], (c) Berlin Mood Questionnaire (Berliner Stimmungsfragebogen; BSF) [4], (d) Perceived Stress Questionnaire (PSQ) [5], (e) Short Form-8 Health Survey (SF8) [6], (f) a sociodemographics questionnaire (SOZK) [7], (g) Self-Efficacy- Optimism-Pessimism Scale (Selbstwirksamkeits-Optimismus-Pessimismus Skala; SWOP) [8], (h) visual analogue scales measuring tinnitus loudness, frequency and distress (TINSKAL) and the (i) Tinnitus Localization and Quality questionnaire (TLQ) [9].

No.	Feature	Description	Response options
1	ADSL_lie	Lie detection score	-
2	ADSL_depression	Depressive disorder sum score	-
3	ADSL_adsl01	"During the past week I was bothered by things that usually don't bother me."	0=rarely; 1=some; 2=occasionally; 3=most
4	ADSL_adsl02	"During the past week I did not feel like eating; my appetite was poor."	same as #3
5	ADSL_adsl03	"During the past week I felt that I could not shake off the blues even with help from my family or friends."	same as #3
6	ADSL_adsl04	"During the past week I felt I was just as good as other people."	0=most; 1=occasionally; 2=some; 3=rarely
7	ADSL_adsl05	"During the past week I had trouble keeping my mind on what I was doing."	same as #3
8	ADSL_adsl06	"During the past week I felt depressed."	same as #3
9	ADSL_adsl07	"During the past week I felt that everything I did was an effort."	same as #3
10	ADSL_adsl08	"During the past week I felt hopeful about the future."	same as #6
11	ADSL_adsl09	"During the past week I thought my life had been a failure."	same as #3
12	ADSL_adsl10	"During the past week I felt fearful."	same as #3
13	ADSL_adsl11	"During the past week my sleep was restless."	same as #3
14	ADSL_adsl12	"During the past week I was happy."	same as #6
15	ADSL_adsl13	"During the past week I talked less than usual."	same as #3
16	ADSL_adsl14	"During the past week I felt lonely."	same as #3
17	ADSL_adsl15	"During the past week people were unfriendly."	same as #3
18	ADSL_adsl16	"During the past week I enjoyed life."	same as #6
19	ADSL_adsl17	"During the past week I had crying spells."	same as #3
20	ADSL_adsl18	"During the past week I felt sad."	same as #3
21	ADSL_adsl19	"During the past week I felt that people disliked me."	same as #3
22	ADSL_adsl20	"During the past week I could not get going."	same as #3
23	ADSL_timestamp	ADSL: avg. time needed to fill an item (in secs)	-
24	BI_complaint	Complaint stress score	-
25	BI_exhaust	Exhaustion score	-
26	BI_gbb01	Exhaustion - feeling of faintness	0=not at all; 1=hardly; 2=somewhat; 3=considerably; 4=severely
27	BI_gbb02	Heart complaint - palpitation, tachycardia or allodromy	same as #26

Continued on next page

No.	Feature	Description	Response options
28	BI_gbb03	Epigastralgia - feeling of pressure or abdominal bloating	same as #26
29	BI_gbb07	Exhaustion - exaggeratedly in need of sleep	same as #26
30	BI_gbb09	Limb pain - joint pain or limb pain	same as #26
31	BI_gbb10	Heart complaint - vertigo	same as #26
32	BI_gbb11	Limb pain - sacroiliac pain or backache	same as #26
33	BI_gbb13	Limb pain - cervicalgia or shoulder pain	same as #26
34	BI_gbb15	Epigastralgia - vomition	same as #26
35	BI_gbb18	Epigastralgia - nausea	same as #26
36	BI_gbb20	Heart complaint - globus sensation, pokiness or gagging	same as #26
37	BI_gbb23	Epigastralgia - ructus	same as #26
38	BI_gbb25	Epigastralgia - heartburn or acid belching	same as #26
39	BI_gbb27	Limb pain - headache	same as #26
40	BI_gbb29	Exhaustion - rapid exhaustibility	same as #26
41	BI_gbb32	Exhaustion - fatigue	same as #26
42	BI_gbb36	Exhaustion - dazed feeling	same as #26
43	BI_gbb41	Limb pain - feeling of heaviness or tiredness in legs	same as #26
44	BI_gbb42	Exhaustion - languor	same as #26
45	BI_gbb45	Heart complaint - stitches, breast pain	same as #26
46	BI_gbb51	Epigastralgia - stomachache	same as #26
47	BI_gbb52	Heart complaint - asthma	same as #26
48	BI_gbb55	Limb pain - feeling of pressure in head	same as #26
49	BI_gbb56	Heart complaint - heart complaint in spasms	same as #26
50	BI_limbs	Limb pain score	-
51	BI_heart	Heart complaint score	-
52	BI_epigastralgia	Epigastralgia score	-
53	BI_timestamp	BI: avg. time needed to fill an item (in secs)	-
54	BSF_anger	Anger score	-
55	BSF_anx_depress	Anxious depressiveness score	-
56	BSF_bsf01	Fatigue - faint	0=not at all; 1=somewhat; 2=rather; 3=mainly; 4=very much
57	BSF_bsf02	Engagement - focused	same as #56
58	BSF_bsf03	Lightened mood - relaxed	same as #56
59	BSF_bsf04	Apathy - indifferent	same as #56
60	BSF_bsf05	Anxious depression - worried	same as #56
61	BSF_bsf06	Anger - irritable	same as #56
62	BSF_bsf07	Fatigue - slack	same as #56
63	BSF_bsf08	Anger - belligerent	same as #56
64	BSF_bsf09	Apathy - uninvolved	same as #56
65	BSF_bsf10	Lightened mood - cheerful	same as #56
66	BSF_bsf11	Fatigue - tired	same as #56
67	BSF_bsf12	Anxious depression - concerned	same as #56
68	BSF_bsf13	Engagement - deliberate	same as #56
69	BSF_bsf14	Anger - edgy	same as #56
70	BSF_bsf15	Lightened mood - buoyant	same as #56
71	BSF_bsf16	Apathy - bored	same as #56
72	BSF_bsf17	Fatigue - haggard	same as #56

Continued on next page

No.	Feature	Description	Response options
73	BSF_bsf18	Engagement - sympathetic	same as #56
74	BSF_bsf19	Anger - angry	same as #56
75	BSF_bsf20	Lightened mood - composed	same as #56
76	BSF_bsf21	Anger - aggressive	same as #56
77	BSF_bsf22	Apathy - apathetic	same as #56
78	BSF_bsf23	Anxious depression - insecure	same as #56
79	BSF_bsf24	Anxious depression - depressed	same as #56
80	BSF_bsf25	Engagement - helpful	same as #56
81	BSF_bsf26	Lightened mood - jolly	same as #56
82	BSF_bsf27	Engagement - considerate	same as #56
83	BSF_bsf28	Apathy - disinterested	same as #56
84	BSF_bsf29	Fatigue - flaccid	same as #56
85	BSF_bsf30	Anxious depression - saddened	same as #56
86	BSF_engagement	Engagement score	-
87	BSF_mood	Lightened mood score	-
88	BSF_fatigue	Fatigue score	-
89	BSF_timestamp	BSF: avg. time needed to fill an item (in secs)	-
90	BSF_apathy	Apathy score	-
91	PSQ_demand	Demand score	-
92	PSQ_tension	Tension score	-
93	PSQ_joy	Joy score	-
94	PSQ_stress	Stress sum score	-
95	PSQ_worries	Worries score	-
96	PSQ_stress01	"You feel rested."	1=hardly ever; 2=rarely; 3=frequently; 4=mostly
97	PSQ_stress02	"You feel that too many demands are being made on you."	same as #96
98	PSQ_stress03	"You are irritable or grouchy."	same as #96
99	PSQ_stress04	"You have too many things to do."	same as #96
100	PSQ_stress05	"You feel lonely or isolated."	same as #96
101	PSQ_stress06	"You find yourself in situations of conflict."	same as #96
102	PSQ_stress07	"You feel you're doing things you really like."	same as #96
103	PSQ_stress08	"You feel tired."	same as #96
104	PSQ_stress09	"You fear you may not manage to attain your goals."	same as #96
105	PSQ_stress10	"You feel calm."	same as #96
106	PSQ_stress11	"You have too many decisions to make."	same as #96
107	PSQ_stress12	"You feel frustrated."	same as #96
108	PSQ_stress13	"You are full of energy."	same as #96
109	PSQ_stress14	"You feel tense."	same as #96
110	PSQ_stress15	"Your problems seem to be piling up."	same as #96
111	PSQ_stress16	"You feel you're in a hurry."	same as #96
112	PSQ_stress17	"You feel safe and protected."	same as #96
113	PSQ_stress18	"You have many worries."	same as #96
114	PSQ_stress19	"You are under pressure from other people."	same as #96
115	PSQ_stress20	"You feel discouraged."	same as #96
116	PSQ_stress21	"You enjoy yourself."	same as #96
117	PSQ_stress22	"You are afraid for the future."	same as #96
118	PSQ_stress23	"You feel you're doing things because you have to not because you want to."	same as #96

Continued on next page

No.	Feature	Description	Response options
119	PSQ_stress24	“You feel criticized or judged.”	same as #96
120	PSQ_stress25	“You are lighthearted.”	same as #96
121	PSQ_stress26	“You feel mentally exhausted.”	same as #96
122	PSQ_stress27	“You have trouble relaxing.”	same as #96
123	PSQ_stress28	“You feel loaded down with responsibility.”	same as #96
124	PSQ_stress29	“You have enough time for yourself.”	same as #96
125	PSQ_stress30	“You feel under pressure from deadlines.”	same as #96
126	PSQ_timestamp	PSQ: avg. time needed to fill an item (in secs)	-
127	SF8_bodily_health	Bodily pain score	-
128	SF8_genhealth	General health score	-
129	SF8_mental	Mental health summary score	-
130	SF8_mentalhealth	Mental health score	-
131	SF8_physicalhealth	Physical health summary score	-
132	SF8_physicalfunc	Physical functioning score	-
133	SF8_emotional	Role emotional score	-
134	SF8_physical	Role physical score	-
135	SF8_social	Social functioning score	-
136	SF8_sf01	“Overall, how would you rate your health during the past week?”	1=excellent; 2=very good; 3=good; 4=fair; 5=poor; 6=very poor
137	SF8_sf02	“During the past 4 weeks, how much did physical health problems limit your physical activities (such as walking or climbing stairs)?”	1=not at all; 2=very little; 3=somewhat; 4=quite a lot; 5=could not do physical activities
138	SF8_sf03	“During the past 4 weeks, how much difficulty did you have doing your daily work, both at home and away from home, because of your physical health?”	1=none at all; 2=a little bit; 3=some; 4=quite a lot; 5=could not do daily work
139	SF8_sf04	“How much bodily pain have you had during the past 4 weeks?”	1=none; 2=very mild; 3=mild; 4=moderate; 5=severe; 6=very severe
140	SF8_sf05	“During the past 4 weeks, how much energy did you have?”	1=very much; 2=quite a lot; 3=some; 4=a little; 5=none
141	SF8_sf06	“During the past 4 weeks, how much did your physical health or emotional problems limit your usual social activities with family or friends?”	1=not at all; 2=very little; 3=somewhat; 4=quite a lot; 5=could not do social activities
142	SF8_sf07	“During the past 4 weeks, how much have you been bothered by emotional problems (...) ?”	1=not at all; 2=slightly; 3=moderately; 4=quite a lot; 5=extremely.
143	SF8_sf08	“During the past 4 weeks, how much did personal or emotional problems keep you from doing your usual work, school or other daily activities?”	1=not at all; 2=very little; 3=somewhat; 4=quite a lot; 5=could not do daily activities
144	SF8_timestamp	SF8: avg. time needed to fill an item (in secs)	-
145	SF8_vitality	Vitality score	-

Continued on next page

No.	Feature	Description	Response options
146	SOZK_gender	Male gender	-
147	SOZK_nationality	German nationality	-
148	SOZK_partnership	In partnership	-
149	SOZK_divorced	Divorced	-
150	SOZK_married	Married	-
151	SOZK_unmarried	Unmarried	-
152	SOZK_abitur	Education level: "Abitur"	-
153	SOZK_fachabitur	Education level: "Fachabitur"	-
154	SOZK_lowersec	Education level: lower secondary school	-
155	SOZK_mittlreife	Education level: "mittlere Reife"	-
156	SOZK_geselle	Education level: "Geselle"	-
157	SOZK_graduate	Education level: university	-
158	SOZK_nograd	Education level: none	-
159	SOZK_meister	Education level: "Meister"	-
160	SOZK_pupil	Education level: currently pupil or "Azubi"	-
161	SOZK_student	Education level: currently student	-
162	SOZK_job	Job status: currently employed	-
163	SOZK_unemp	Duration of unemployment in the last 5 years	-
164	SOZK_employee	Occupation: employee	-
165	SOZK_worker	Occupation: worker	-
166	SOZK_civservant	Occupation: civil servant	-
167	SOZK_occupother	Occupation: other	-
168	SOZK_selfempl	Occupation: self-employed	-
169	SOZK_ill	"How long have you been ill during the last 12 months? (in months)"	-
170	SOZK_tinnitusdur	"How long have you been suffering from tinnitus (in years)?"	-
171	SOZK_psychotreat	"How long have you been in psychotherapeutic treatment? (in months)"	-
172	SOZK_nophysicians	"How many physicians have you visited because of your current complaints?"	-
173	SOZK_timestamp	SOZK: avg. time needed to fill an item (in secs)	-
174	SWOP_optimism	Optimism score	-
175	SWOP_pessimism	Pessimism score	-
176	SWOP_selfefficacy	Self-efficacy score	-
177	SWOP_swo01	"If someone makes a stand against me, I will find ways to prevail."	1=not true; 2=hardly true; 3=rather true; 4=very true
178	SWOP_swo02	"I almost never expect things to go my way."	same as #177
179	SWOP_swo03	"I have no trouble realizing my intentions and goals."	same as #177
180	SWOP_swo04	"I am always optimistic about the future."	same as #177
181	SWOP_swo05	"I always know how to behave in unexpected situations."	same as #177
182	SWOP_swo06	"Things never develop as I wish."	same as #177
183	SWOP_swo07	"Even with surprising events, I believe that I will get along well with it."	same as #177
184	SWOP_swo08	"I can easily face difficulties because I can always rely on my abilities."	same as #177
185	SWOP_swo09	"I always see the good side of things."	same as #177
186	SWOP_timestamp	SWOP: avg. time needed to fill an item (in secs)	-

Continued on next page

No.	Feature	Description	Response options
187	TINSKAL_impairment	Degree of tinnitus impairment	-
188	TINSKAL_frequency	Tinnitus frequency	-
189	TINSKAL_loudness	Tinnitus loudness	-
190	TINSKAL_timestamp	TINSKAL: avg. time needed to fill an item (in secs)	-
191	TLQ_timestamp	TLQ: avg. time needed to fill an item (in secs)	-
192	TLQ_rightear	The tinnitus is located in the right ear.	-
193	TLQ_leftear	The tinnitus is located in the left ear.	-
194	TLQ_bothears	The tinnitus is located in both ears.	-
195	TLQ_entirehead	The tinnitus is located in the entire head.	-
196	TLQ_whistling	Tinnitus noise: whistling	-
197	TLQ_hissing	Tinnitus noise: hissing	-
198	TLQ_ringing	Tinnitus noise: ringing	-
199	TLQ_rustling	Tinnitus noise: rustling	-
200	age	patient age	-
201	testing_day	testing date: day of the month	-
202	testing_month	testing date: month	-
203	testing_week	testing date: calendar week	-
204	testing_weekday	testing date: day of the week	-
205	testing_yearday	testing date: day of the year	-

References

1. Radloff LS. The CES-D scale: a self-report depression scale for research in the general population. Applied psychological measurement. 1977;1(3):385–401.
2. Hautzinger M, Bailer M. ADS-Allgemeine Depressionsskala. In: Diagnostische Verfahren in der Psychotherapie. Beltz; 2003.
3. Hörhold M, Bolduan D, Klapp C, Volger H, Scholler G, Klapp B. Testing a screening strategy for identifying psychosomatic patients in gynecologic practice. Psychotherapie, Psychosomatik, medizinische Psychologie. 1997;47(5):156–162.
4. Hörhold M, Klapp B, Schimmack U. Testungen der Invarianz und der Hierarchie eines mehrdimensionalen Stimmungsmodells auf der Basis von Zweipunkterhebungen an Patienten-und Studentenstichproben. Z med Psychol. 1993;2(1):27–35.
5. Fliege H, Rose M, Arck P, Walter OB, Kocalevent RD, Weber C, et al. The Perceived Stress Questionnaire (PSQ) reconsidered: validation and reference values from different clinical and healthy adult samples. Psychosomatic medicine. 2005;67(1):78–88.
6. Bullinger M, Morfeld M. Der SF-36 Health Survey. In: Gesundheitsökonomische Evaluationen. Springer; 2008. p. 387–402.
7. Brüggemann P, Szczepek AJ, Rose M, McKenna L, Olze H, Mazurek B. Impact of multiple factors on the degree of tinnitus distress. Frontiers in human neuroscience. 2016;10:341.
8. Scholler G, Fliege H, Klapp BF. Fragebogen zu Selbstwirksamkeit, Optimismus und Pessimismus. Psychother Psychosom Med Psychol. 1999;49(8):275–283.
9. Goebel G, Hiller W. Psychische Beschwerden bei chronischem Tinnitus: Erprobung und Evaluation des Tinnitus-Fragebogens (TF). Verhaltenstherapie. 1992;2(1):13–22.
