# Supplementary material for: Tinnitus-related distress after multimodal treatment can be characterized using a key subset of baseline variables
Source: PLoS One. 2020 Jan 30;15(1):e0228037. doi: 10.1371/journal.pone.0228037 (PMC6991951; doi:10.1371/journal.pone.0228037)
Supplement: S2 Table — The potential of machine learning for the prediction of TQ_distress at T1 (after treatment) using questionnaire data from T0 only was investigated with the following 11 algorithms: LASSO [25], RIDGE [26], support vector machine (SVM) [28], a feed-forward neural network with one single hidden layer (NNET) [29], generalized partial least squares (GPLS) [27], weighted k-nearest neighbor classifier (WKNN) [30], Naïve Bayes classifier (NB), CART decision tree [31], C5.0 decision tree [32], random forest (RF) [33] and gradient boosted trees (GBT) [34]. All classifiers were implemented with the statistical programming language R [44] using the package mlr [45], which provides a consistent interface to many machine learning algorithms from other R packages. A grid search was employed for hyperparameter tuning using area under the ROC curve (AUC) as evaluation measure. The table below provides an overview about each classifier, including used R package, tuned hyperparameters and their value ranges. Any other hyperparameters were set to default values. (PDF) [file pone.0228037.s002.pdf]

S2 Table. Classifier hyperparameter tuning grid. The potential of machine learning for the prediction of TQ_distress at T1 (after treatment) using questionnaire data from T0 (at baseline) only was investigated with the following 11 algorithms: LASSO [1], RIDGE [2], support vector machine (SVM) [3], a feed-forward neural network with one single hidden layer (NNET) [4], generalized partial least squares (GPLS) [5], weighted k-nearest neighbor classifier (WKNN) [6], Naïve Bayes classifier (NB), CART decision tree [7], C5.0 decision tree [8], random forest (RF) [9] and gradient boosted trees (GBT) [10]. All classifiers were implemented with the statistical programming language R [11] using the package mlr [12], which provides a consistent interface to many machine learning algorithms from other R packages. A grid search was employed for hyperparameter tuning using area under the ROC curve (AUC) as evaluation measure. The table below provides an overview about each classifier, including used R package, tuned hyperparameters and their value ranges. Any other hyperparameters were set to default values.

Algorithm (R package)	Hyperparameter	Min	Max	#Values
lasso, ridge (both glmnet [1])	lambda	0.01	10^{10}	100
wknn (kknn [6])	k	1	77	20
svm (e1071 [13])	cost	0.01	3	6
	gamma	0	3	4
	kernel	*	-	4
nb (e1071 [13])	laplace	1	5	5
nnet (nnet [14])	size	1	13	7
	decay	10^{-4}	1	6
gpls (caret [15])	ncomp	1	5	5
cart (rpart [16])	cp	0.001	0.1	5
c5.0 (C50 [17])	CF	0	0.35	7
	rules	FALSE	TRUE	2
	winnow	FALSE	TRUE	2
rf (ranger [18])	mtry	4	100	7
	min.node.size	1	25	6
gbt (xgboost [19])	eta	0.01	0.4	4
	max_depth	1	3	3
	colsample_bytree	0.2	1	5
	min_child_weight	0.5	2	3
	subsample	0.2	1	3
	nrounds	50	250	3

* = {linear, polynomial, radial, sigmoid}

References

1. Friedman J, Hastie T, Tibshirani R. Regularization Paths for Generalized Linear Models via Coordinate Descent. Journal of Statistical Software. 2010;33(1):1–22.
2. Hoerl AE, Kennard RW. Ridge regression: Biased estimation for nonorthogonal problems. Technometrics. 1970;12(1):55–67.
3. Boser BE, Guyon IM, Vapnik VN. A training algorithm for optimal margin classifiers. In: Proc. of Workshop on Computational Learning Theory. ACM; 1992. p. 144–152.
4. Venables WN, Ripley BD. Modern Applied Statistics with S. 4th ed. Springer; 2002.
5. Ding B, Gentleman R. Classification using generalized partial least squares. Journal of Computational and Graphical Statistics. 2005;14(2):280–298.
6. Hechenbichler K, Schliep K. Weighted k-Nearest-Neighbor Techniques and Ordinal Classification. In: SFB 386, Ludwig-Maximilians University, Munich. vol. 399 of sfb386; 2004.Available from: `http://nbn-resolving.de/urn/resolver.pl?urn=nbn:de:bvb:19-epub-1769-9`.
7. Breiman L, Friedman J, Olshen R, Stone C. Classification and Regression Trees. Wadsworth and Brooks; 1984.
8. Quinlan R. C4.5: Programs for Machine Learning. San Mateo, CA: Morgan Kaufmann Publishers; 1993.
9. Breiman L. Random forests. Machine learning. 2001;45(1):5–32.
10. Friedman JH. Greedy function approximation: a gradient boosting machine. Annals of Statistics. 2001; p. 1189–1232.
11. R Core Team. R: A Language and Environment for Statistical Computing; 2018. Available from: `https://www.r-project.org/`.
12. Bischl B, Lang M, Kotthoff L, Schiffner J, Richter J, Studerus E, et al. mlr: Machine Learning in R. The Journal of Machine Learning Research. 2016;17(1):5938–5942.
13. Dimitriadou E, Hornik K, Leisch F, Meyer D, Weingessel A. e1071: Misc Functions of the Department of Statistics (e1071), TU Wien. R package version 1.5-25.; 2011. Available from: `http://cran.r-project.org/package=e1071`.
14. Venables WN, Ripley BD. Modern Applied Statistics with S. 4th ed. New York: Springer; 2002. Available from: `http://www.stats.ox.ac.uk/pub/MASS4`.
15. Kuhn M. Building Predictive Models in R Using the caret Package. Journal of Statistical Software, Articles. 2008;28(5):1–26. doi:10.18637/jss.v028.i05.
16. Therneau T, Atkinson B. rpart: Recursive Partitioning and Regression Trees; 2018. Available from: `https://CRAN.R-project.org/package=rpart`.
17. Kuhn M, Quinlan R. C50: C5.0 Decision Trees and Rule-Based Models; 2018. Available from: `https://CRAN.R-project.org/package=C50`.
18. Wright MN, Ziegler A. ranger: A Fast Implementation of Random Forests for High Dimensional Data in C++ and R. Journal of Statistical Software. 2017;77(1):1–17. doi:10.18637/jss.v077.i01.

19. Chen T, Guestrin C. XGBoost: A Scalable Tree Boosting System. In: Proc. of ACM SIGKDD International Conference on Knowledge Discovery and Data Mining. New York, NY, USA: ACM; 2016. p. 785–794. Available from: http://doi.acm.org/10.1145/2939672.2939785.
