# Supplementary material for: Tinnitus-related distress after multimodal treatment can be characterized using a key subset of baseline variables
Source: PLoS One. 2020 Jan 30;15(1):e0228037. doi: 10.1371/journal.pone.0228037 (PMC6991951; doi:10.1371/journal.pone.0228037)
Supplement: S3 Table — Relative frequencies are given in percent. (PDF) [file pone.0228037.s003.pdf]

S3 Table. Comparison of completer and non-completer characteristics. Relative frequencies are given in percent.

Characteristic	Completer N=1,416	Non-Completer N=2,701
Age		
12 – 19	0.5	1.0
20 – 29	6.7	7.3
30 – 39	13.6	10.3
40 – 49	23.8	22.4
50 – 59	34.5	29.0
60 – 69	16.6	19.5
70 – 79	4.2	9.7
80 – 89	0.0	0.7
90	-	0.0
N/A	-	-
Gender		
female	50.9	42.0
male	49.1	50.0
N/A	-	8.0
Years since tinnitus onset		
≤ 0.5	13.7	22.5
0.5 – 1	20.5	17.8
1 – 2	15.7	13.1
2 – 5	16.7	13.4
> 5	33.3	25.1
N/A	-	8.0
Education level		
Abitur (general qualification for university entrance)	48.0	37.9
Fachabitur (qualification for university of applied sciences entrance)	11.6	8.3
Mittlere Reife ($\approx$ high school)	28.9	29.4
Hauptschule (lower secondary education)	11.1	14.6
other	0.4	1.8
N/A	-	8.0
